# Identification of Rare PB2-D701N Mutation from a Patient with Severe Influenza: Contribution of the PB2-D701N Mutation to the Pathogenicity of Human Influenza

**DOI:** 10.3389/fmicb.2017.00575

**Published:** 2017-04-03

**Authors:** Amelia Nieto, Francisco Pozo, Matxalen Vidal-García, Manuel Omeñaca, Inmaculada Casas, Ana Falcón

**Affiliations:** ^1^Centro Nacional de Biotecnología – Consejo Superior de Investigaciones CientíficasMadrid, Spain; ^2^Ciber de Enfermedades Respiratorias (CIBERES), MadridSpain; ^3^National Influenza Center, Centro Nacional de Microbiología, Instituto de Salud Carlos IIIMadrid, Spain; ^4^Servicio de Microbiología, Hospital Universitario Miguel ServetZaragoza, Spain

**Keywords:** influenza virus, pathogenicity, PB2 subunit, D701N mutation, adaptation of avian viruses

## Abstract

Several amino acid changes have been previously implicated in adaptation of avian influenza viruses to human hosts, among them the D701N change in the PB2 polymerase subunit that also is the main determinant of avian virus pathogenesis in animal models. However, previous studies using recombinant viruses did not provide conclusive information of the contribution of this PB2 residue to pathogenicity in human influenza virus strains. We identified this mutation in an A(H1N1)pdm09-like human influenza virus isolated from an infected patient with pneumonia and acute respiratory failure, admitted to the intensive care unit. An exhaustive search has revealed PB2-D701 as a highly conserved position in all available H1N1 human virus sequences in NCBI database, showing a very low prevalence of PB2-D701N change. Presence of PB2-701N amino acid correlates with severe or fatal outcome in those scarce cases with known disease outcome of the infection. In these patients, the residue PB2-701N may contribute to pathogenicity as it was previously reported in humans infected with avian viruses. This study helps to clarify a debate that has arisen regarding the role of PB2-D701N in human influenza virus pathogenicity.

## Influenza Virus

Acute respiratory infections continue to be the main cause of acute illnesses worldwide and influenza virus is one of the major contributors. The average global burden seasonal influenza comes to be more than 600 million cases, 3 million cases of severe illness and 300,000–500,000 deaths per year worldwide^1^. Influenza A belongs to the family of negative stranded, segmented RNA viruses known as Orthomyxoviridae, being the pathogens of major concern in veterinary and public health. In their natural zoonotic reservoirs, the viruses are largely benign, causing asymptomatic enteric infection in the wild aquatic birds (Webster et al., 1992), from here influenza A viruses spread to domestic poultry, pigs, or people and they can evolve into strains that cause mild to catastrophic disease.

Influenza virus possesses a negative-sense single-stranded RNA genome divided into eight segments. The polymerase subunits (PA, PB1 and PB2) and the nucleoprotein (NP) are responsible for genome expression. These proteins associate to each viral RNA segment to constitute the viral ribonucleoproteins (Elton et al., 2005; Resa-Infante et al., 2010) that form the minimum set of viral proteins required for viral RNA transcription and replication (reviewed in Resa-Infante et al., 2011; Fodor, 2013). The genome of influenza virus encodes for 10 major proteins, although the virus uses different mechanisms to express alternative protein products from several genome segments. These include alternative splicing of viral mRNAs or non-canonical translation strategies. Ten viral proteins have been studied extensively but five more proteins have recently been identified, which would increase the degree of viral replication control (Vasin et al., 2014).

Influenza A virus pathogenesis is a complex multigenic mechanism, and pathogenic determinants are distributed throughout the genome but, these determinants map mainly to the polymerase genes, the surface glycoproteins hemagglutinin (HA) and neuraminidase (NA) that are the targets of antibodies that block the infection, and non-structural protein 1 (NS1), a multifunctional protein that has a major role counteracting the antiviral response (reviewed in Medina and Garcia-Sastre, 2011; Rodriguez-Frandsen et al., 2015).

## Transmission of Avian Influenza Viruses to Mammals: Implications in Pathogenicity

Most influenza A viruses infecting avian species in nature are non-pathogenic but a few caused by strains of subtypes H5 and H7 are highly pathogenic to poultry and cause fulminating disease with mortality close to 100% (Swayne and Pantin-Jackwood, 2006; Capua and Alexander, 2007; Franca and Brown, 2014). Transmission of these highly pathogenic H5 and H7 avian influenza viruses to humans is not very frequent, although the resulting disease is often severe or fatal (Claas et al., 1998; Subbarao et al., 1998; Li et al., 2004; Horimoto and Kawaoka, 2005; Yen and Webster, 2009).

When infecting a new host influenza virus needs to accomplish three crucial steps for successful infection and propagation: (i) entry into the host cell, (ii) efficient replication of the viral genome, and (iii) dissemination of progeny virions into a new host (Sorrell et al., 2011). Preferential interaction of the HA with different sialic acid cell receptors constitutes an important host range determinant for influenza viruses (reviewed in de Graaf and Fouchier, 2014). In addition, several described mutations in the viral polymerase subunits are known to enhance virus replication in mammalian cells and play an essential role in virus adaptability to various hosts (Rodriguez-Frandsen et al., 2015).

Analyzed H5 and H7 isolates from humans have mutations involved in adaptation to the new host (Herfst et al., 2014; Watanabe et al., 2014). Many of these are located in the polymerase PB2 subunit, with E627K and D701N as the most prevalent mutations (Subbarao et al., 1993; Hatta et al., 2001; Steel et al., 2009; Chen et al., 2013; Gabriel et al., 2013; Jonges et al., 2014), which are rarely present in human circulating viruses. These changes in PB2 are also implicated in pathogenesis in animal models. In H5N1 viruses isolated from ducks in China, PB2-701N was the main pathogenicity determinant in the mouse model (Shinya et al., 2004; Li et al., 2005), and also partly determined the high pathogenicity of an A/seal/Massachusetts/1/1980 (H7N7) virus (Gabriel et al., 2005). In addition, experiments with recombinant viruses using the A/Anhui/1/2013 avian H7N9 strain as a backbone and bearing PB2-701N or PB2-701D showed higher viral titers for PB2-701N than PB2-701D recombinant viruses in human A549-infected cells and mouse lungs. Viral polymerase activity bearing PB2-701N in avian viruses was also increased in reconstitution experiments in human A549 cell line (Yamayoshi et al., 2015). These data indicate that residue PB2-701N contributes to augment pathogenesis in human cells and mice infected with avian isolates.

Few protein residues from the PB1 polymerase protein have been connected to host adaptation of influenza viruses. PB1 has a role in pathogenicity and polymerase activity and two mutations in PB1, PB1-L473V and L598P, compensated for the absence of PB2-627K within an attenuated reassortant virus containing the H5N1 polymerase in a WSN background, both in cell culture and in the mouse model (Xu et al., 2012). The minimal set of mutations required for airborne transmission of a H5N1 virus among ferrets included, in addition of the PB2-E627K change, mutation PB1-H99Y (Linster et al., 2014), both mutations together had a synergistic effect, increasing viral polymerase activity and virus replication in mammalian cells.

PA subunit has been recognized as a key factor modulating viral polymerase activity and pathogenicity in *in vitro* and *in vivo* (reviewed in Rodriguez-Frandsen et al., 2015). Several mutations in PA have been linked to enhanced virulence, including G631S in an H5N1 avian virus (Hiromoto et al., 2000), or N409S in the novel avian-origin H7N9 virus that may confer higher replication in mammals (Yamayoshi et al., 2014). Mutations PA T97Y (Mehle et al., 2012) and T552S (Song et al., 2009) are also involved in an increased viral replication and pathogenicity of avian viruses in mice.

## PB2 Segment of Influenza A(H1N1)pdm09 Virus

The influenza A virus from the 2009 pandemic, A(H1N1)pdm09 virus, has segments of avian, human and porcine origin. This virus showed high transmissibility, but relatively low virulence, spread rapidly across the globe and caused the first pandemic of the 21st century (Neumann et al., 2009). A(H1N1)pdm09 virus has an avian-lineage PB2 gene that lacks the E627K and D701N substitutions that permit transmission and cooperate in pathogenesis of avian-origin influenza viruses in humans or other mammals. This virus nonetheless had a compensatory PB2-Q591R substitution that rendered PB2-E627K unnecessary for effective replication (Mehle and Doudna, 2009).

Several reverse genetics studies have tested whether mutation of the PB2 polymerase at residue 701 enhances virulence in human A(H1N1)pdm09 viruses and obtained contradictory results. Recombinant viruses were generated using the pandemic A/Netherlands/602/2009 strain as a backbone. Enhanced polymerase activity was found by mini-genome analysis using a PB2 segment bearing PB2-701N compared with PB2-701D. When mice and ferrets were infected with these two recombinant viruses, PB2-701N-infected ferrets showed greater body weight loss, although the virus with this mutation caused no other major differences in global pathogenesis in mice and ferrets (Herfst et al., 2010).

A different study used reassortant viruses containing the four RNP genes (PB1, PB2, PA and NP genes) of the representative A/California/04/2009 pandemic virus on the background of the remaining four gene segments from the A/New York/312/2001 (H1N1) seasonal human influenza virus. In human cell cultures and in mice, the reassortant virus containing PB2-701N substitution was attenuated (Jagger et al., 2010). In contrast, in a more defined recombinant virus using the pdm09-lineage A/New York/1682/2009 strain as backbone and having PB2-701D or PB2-701N, higher replication rates in human A549 cells and increased polymerase activity in a mini-genome replication assay was found for the virus with PB2-701N (Zhou et al., 2013). Moreover, the PB2-701N virus was more pathogenic in the mouse model and showed more efficient transmission in ferrets (Zhou et al., 2013). These three reports do not provide conclusive results regarding the contribution of PB2 residue 701 in human influenza virus strains, and suggest distinct outcomes depending on genetic background.

## Identification of 701N in PB2 Subunit of an Influenza Virus Isolated From an Infected Patient Admitted to Intensive Care Unit

In a search for specific changes in the polymerase of influenza viruses that might contribute to viral pathogenicity in infected patients, we evaluated the viruses present in the pharyngeal exudate of patients diagnosed with influenza AH1N1pdm09-linage infection, under 65 years of age admitted to the intensive care unit (ICU) during the Spanish 2013–2014 epidemic season. Respiratory samples were collected and delivered to the Spanish National Influenza Center (CNM, ISCIII) for virological characterization and virus isolation.

PB1, PB2 and PA segments sequences of all viruses were obtained from Sanger sequencing of RT-PCR products generated from the original clinical samples. We found a virus bearing the PB2-D701N mutation (A/Aragon/270/2014) in the clinical sample of one patient who met the above mentioned requirements. The patient was a 60-year-old man admitted to the hospital with onset of fever, asthenia, and cough persisted for 3 days. Past medical history included type 2 diabetes as comorbid condition. The progressive deterioration of clinical conditions with worsening wheezing, increased secretions and progressive dyspnea at rest determined the admission to ICU for monitoring and treatment. The patient was diagnosed with pneumonia and acute respiratory failure after analytical test that displayed a remarkable thrombocytopenia (71 × 10^3^cells/μl) and chest radiography showing increased density in lower left lung field. After 3 days at the ICU with high flow nasal oxygenation (>21%), with oseltamivir, ceftriaxone and azithromycin treatment the patient showed good evolution and was discharged 8 days later.

The analysis of the amino acid sequence of the polymerase subunits showed that it had arginine (R) and glutamic acid (E) at PB2 positions 591 and 627, respectively, which coincides with the consensus sequence of the 2009 pandemic strains (**Table 1**, upper).

**Table 1:** Characteristics of the Influenza A H1N1 viruses detected in infected patients.

Human H1N1 Isolates	PB2 591	PB2 627	PB2 701	Clinical manifestations
A/Aragon/270/2014	R	E	N	Pneumonia and acute respiratory failure (ICU)^#^
A/Uganda/MUWRP-111/2009	R	E	N	Unknown
A/Wisconsin/51/2009	R	E	N	Unknown
A/Singapore/GP3828/2010	R	E	N	Unknown
Most A(H1N1)pdm09	R	E	D	Mostly mild

**Human H1N1 isolates from swine origin**	**PB2 591**	**PB2 627**	**PB2 701**	**Clinical manifestations**

A/Jiangsu/ALS1/2011	Q	E	N	Fatal
A/Jiangsu/1/2011	Q	E	N	Unknown
A/Switzerland/9356/2009	Q	T	N	Unknown
A/Switzerland/5165/2010	Q	E	N	Unknown

*^#^Requiring admission to Intensive care unit.*

## Association of PB2-701N With Viral Pathogenicity in the Infected Patient

Primary viral isolation was performed for further genome analysis of this virus. Virus was amplified by one passage in MDCK cells at low multiplicity of infection using the titrated virus isolated from the original clinical sample. Total viral RNA was isolated from purified virions as previously described (Rodriguez et al., 2013), and full genome sequence was determined by next-generation sequencing with TruSeq v3 chemistry and 50 bp single reads on an Illumina HiSeq 2000. FASTQ sequences were aligned against the influenza (A/California/04/09) genome. To determine the consensus sequence of the virus, coverage and nucleotide composition of aligned reads were analyzed. Nucleotide positions with an identity of ≥75% were considered. Samtools mpileup (Li et al., 2009) and in-house php scripts were used.

The full viral genome sequencing allowed the comparison of the complete amino acid sequence of this virus A/Aragon/270/2014 with mild-case viruses from the same influenza season isolated in the sentinel medical centers. The comparison showed three amino acid changes; PB2-D701N, HA-K403R and NA-L415M. A search in the NCBI Influenza Virus Resource database^2^ indicated that NA-L415M change has a prevalence >1% in human influenza A(H1N1)pdm09 virus NA proteins, whereas PB2-D701N and HA-K403R had a prevalence between 0.05 and 0.10%. The PB2-D701N change was present in 100% of the A/Aragon/270/2014 viral sequence reads, but change HA-K403R was present in 85% of total reads. The remaining 15% of the total reads corresponded to the nucleotide present in mild-case circulating viruses.

The K403R HA change is not described as a pathogenicity determinant of those reported for A(H1N1)pdm09 (Safronetz et al., 2011; Martinez-Romero et al., 2013) or H5N1 viruses (Hulse et al., 2004; Heider et al., 2015). In addition, all the described positions in the introduction section, which may mutate to increase viral pathogenicity in mammals, are identical to those of the mild-case human viruses. This data suggests that the previously described PB2-D701N change might be in this patient, the main viral contributor to severe outcome of the infection.

## Low Prevalence of Influenza PB2-701N Virus in Humans

An exhaustive examination of NCBI database showed that only 8 out of 7562 (prevalence 0.105%) PB2 segment sequences detected in H1N1 virus infected patients contained a different amino acid than PB2-D701 from April 1950 until March 2016 (**Tables 1**, **2**), indicating a highly conservancy of this position in H1N1 virus detected in humans. Seven of them bore PB2-D701N change (eight, including the viral isolate here described, prevalence 0.105%), and corresponded to human A(H1N1)pdm09-linage isolates obtained from the start of the 2009 pandemic until March 2016. Comparison of the HA sequences of these isolates indicated that four had a swine origin HA gene, and thus corresponded to humans infected directly with porcine viruses (0.052%) (**Table 1** lower, **Table 2**). The death 40 days post-infection of a 3-year-old child was the unique report among these patients infected with porcine strains (Qi et al., 2013). Sequence analysis of the other three human isolates containing PB2-D701N mutation indicated that their HA protein was the human protein present among circulating A(H1N1)pdm09-linage viruses (**Table 1**, upper). Thus, the total prevalence of H1N1 viruses of human origin containing PB2-701N from April 1950 until March 2016, including A/Aragon/270/2014, is four from a total of 7562 (0.052%) available sequences in the NCBI database, and all of them are of A(H1N1)pdm09-linage (**Table 2**). The genome of A/Aragon/270/2014 has been deposited in the NCBI database under accession no. “Pending.” The NCBI database accession numbers of the viral isolates described in this study are indicated in Supplementary Table 1.

**Table 2:** Prevalence, origin, and disease outcome data related to H1N1 viruses bearing changes at position 701 of influenza PB2 polymerase subunit.

PB2-D701 position	Total H1N1 human isolates (%)	Swine origin H1N1 human isolates (%)	A(H1N1)pdm09-linage human isolates (%)	Cases with known disease outcome (%)	Severe/fatal outcome (%)
N amino acid change	8^a^/7562 (0.105)	4/7562 (0.052)	4/7562 (0.052)	2/8 (25)	2/2 (100)
Other amino acid change	1^b^/7562 (0.013)	0/7562 (0)	1/7562 (0.013)	0/1 (0)	0/1 (0)

*^a^: Including viral isolate sequences available in Influenza Resource Database and viral isolate described in this study. ^b^: Amino acid change PBZ-D701Y.*

To determine the spread in the community of A/Aragón/270/2014 virus found in the infected patient admitted to ICU, we further sequenced PB2 segments of 13 human A(H1N1)pdm09-linage viruses detected from positive influenza cases studied during the same influenza season (2013–2014) and the same geographical region. All 13 influenza samples bore PB2-701D, indicating that PB2-701N influenza viruses were not commonly circulating in the area, and remarkably the only case here described containing PB2 D701N mutation was associated with admission to ICU.

## Discussion

Only four influenza A H1N1 human viruses containing glutamine at position 701 of the PB2 polymerase subunit are reported in the Influenza Virus Resource database, which indicates that sporadic mutation at this position is very infrequent (**Table 2**). Among them, the unique virus characterized (A/Aragon/270/2013) was isolated from a patient aged under 65 with pneumonia and acute respiratory failure, admitted to ICU. Although the importance of the HA-K403R change also present in this virus has not been previously characterized for pathogenesis of human influenza strains (Hulse et al., 2004; Safronetz et al., 2011), this mutation was identified in a human influenza A H1N1 virus of swine origin (A/Saskatchewan/5351/2009), which produced mild symptoms in the infected patient (Bastien et al., 2010). These data suggest that the previously described PB2-D701N change may be the main virulence determinant of this viral isolate.

A secondary pneumococcal infection following influenza has been detected in this patient. Influenza virus infection can induce an immunosuppressed state, which is associated with increased incidence of bacterial infections such as *S. pneumoniae*. It has been described that resident macrophage population declines as influenza progresses (reviewed in Smith and McCullers, 2014). Alveolar macrophages play a central role in bacterial establishment, growth, and eradication as revealed in a model analysis. The dose-dependent invasive ability of pneumococci was solely dependent on the number and phagocytic ability of resident macrophages initially present (Smith and McCullers, 2014). In addition, virus-mediated patches of desquamated epithelium allow bacteria to adhere and invade with increase vigor (reviewed in Mina and Klugman, 2014). Thereby, infection with a particularly virulent influenza strain would exacerbate these processes that together with the respiratory tract damage may enhance the subsequent opportunistic bacterial infections.

Regarding the underlying medical conditions of the A/Aragon/270/2013 infected patient, past medical history included type 2 diabetes. It needs to be considered that diabetes, is among the comorbidities involved in a severe outcome of influenza virus infection and it triples the risk of hospitalization after influenza A(H1N1)pdm09 infection (Jimenez-Garcia et al., 2013). However, the results of multivariate analysis have suggested that possibly concomitant conditions such as obesity, frequently found among diabetic persons, highly increases the risk of very severe infection or even death, and not only diabetes itself (Jimenez-Garcia et al., 2013). The patient from whom A/Aragón/270/2014 virus was isolated did not have obesity and his levels of glycated hemoglobin were within the levels of the control population indicating a good control of blood glucose levels upon infection. In addition, during the last 12 years previous to the influenza virus infection here described it does not appear in the clinical history of the patient important infections or reasons for medical consultation attributable to the diabetes. Nevertheless, it cannot be excluded a contribution of this factor to the infection outcome.

Human infections with swine influenza viruses appear to occur infrequently (Schrauwen and Fouchier, 2014) and scarce swine-origin influenza viruses have not been previously associated with human severe/fatal outcome of infection (CDC, 2013). Only four patients have been described infected with swine origin viruses bearing PB2-701N. The infection outcome was reported in only one of them and it was a deceased patient (**Table 1**, lower) (Qi et al., 2013). The analysis of the deceased-case virus sequence showed that amino acids at positions above described other than PB2 701N, whose mutations have been related to increased pathogenicity in mammals, are similar to those of mild-case human viruses and A/Aragon/270/2014 virus. This data suggests that these positions are not contributing to an increased pathogenicity of this virus. Thereby, although other viral or host determinants may have cooperated to this outcome, once again these data point out that PB2-701N amino acid may contribute to a severe or fatal outcome of the infection in humans.

As indicated above the PB2-D701N change produces increased polymerase activity in mini-replicon assays in the context of A(H1N1)pdm09 polymerase. More active polymerase would support a higher viral replication and consequently potential increased pathogenicity. However, PB2-D701N mutation is very scarcely found suggesting that it does not circulate in humans, but that it may be generated in some infected patients, probably as the result of particular virus–host interactions that allow the growth and establishment of the virus carrying this mutation.

An important question is why a virus with a potential increased replication does not spread among human population. One possibility is that viruses containing this mutation have high replication rates but have poor transmission ability among humans, in contrast to the high transmissibility rate that this mutation confers to avian strains for human infections. A different scenario for the barely dispersion of the PB2-N701 virus could be related with its increased virulence. It is possible that patients infected with the virus, are most of the time admitted to the ICU as a consequence of a severe infection outcome, which results in the confinement of the virus reducing its dissemination.

In summary, the data indicate that an Asparagine at position 701 of influenza PB2 polymerase subunit could be contributing to the viral pathogenicity in humans. Future screening of virus containing this change could be crucial for final establishment of this relation.

## Ethics Statement

The National Influenza Center in Madrid (Instituto de Salud Carlos III) and other regional laboratories from different Spanish regions, constituted the ReLEG network included in the Spanish Influenza Surveillance System (SISS), which monitored the circulation of influenza viruses each influenza season as a part of the countrywide surveillance. The virus described in this study has been detected within this surveillance activity. An informed consent is not needed for this study since the patient from whom this virus was isolated was anonymized.

## Author Contributions

AN designed the research studies, conducted databases research and wrote the manuscript. FP selected, isolated and provided the viral isolates and all clinical information, and conducted the experiments. MVG selected, isolated and provided the viral isolates and all clinical information. MO selected, isolated and provided the viral isolates and all clinical information. IC selected, isolated and provided the viral isolates and all clinical information. AF designed the research studies, conducted the experiments, analyzed the data and wrote the manuscript.

## Conflict of Interest Statement

The authors declare that the research was conducted in the absence of any commercial or financial relationships that could be construed as a potential conflict of interest.

## Footnotes

1 http://www.who.int

2 http://www.ncbi.nlm.nih.gov/genomes/FLU/Database/nph-select.cgi
