# Corrigendum: Identification of Rare PB2-D701N Mutation from a Patient with Severe Influenza: Contribution of the PB2-D701N Mutation to the Pathogenicity of Human Influenza

**DOI:** 10.3389/fmicb.2017.01080

**Published:** 2017-06-15

**Authors:** Amelia Nieto, Francisco Pozo, Matxalen Vidal-García, Manuel Omeñaca, Inmaculada Casas, Ana Falcón

**Affiliations:** ^1^Centro Nacional de Biotecnología - Consejo Superior de Investigaciones CientíficasMadrid, Spain; ^2^Ciber de Enfermedades RespiratoriasMadrid, Spain; ^3^National Influenza Center, Centro Nacional de Microbiología, Instituto de Salud Carlos IIIMadrid, Spain; ^4^Servicio de Microbiología, Hospital Universitario Miguel ServetZaragoza, Spain

**Keywords:** Influenza virus, pathogenicity, PB2 subunit, D701N mutation, adaptation of avian viruses

In the original article, there was a mistake in Supplementary Table 1 as published. The first entry (A/Aragon/270/2014) had the Identification number pending. The corrected Supplementary Table 1 has been updated on the original article with the Identification number KY887997 (GISAID EPI_ISL_257783).

In the original article, there was an error. A sentence regarding methodology was missing.

A correction has been made to ASSOCIATION OF PB2-701N WITH VIRAL PATHOGENICITY IN THE INFECTED PATIENT section, Paragraph 1:

Primary viral isolation was performed for further genome analysis of this virus. Virus was amplified by one passage in MDCK cells at low multiplicity of infection using the titrated virus isolated from the original clinical sample. Total viral RNA was isolated from purified virions as previously described (Rodriguez et al., 2013), and full genome sequence was determined by next-generation sequencing with TruSeq v3 chemistry and 50 bp single reads on an Illumina HiSeq 2000. **FASTQ sequences were aligned against the influenza (A/California/04/09) genome**. To determine the consensus sequence of the virus, coverage and nucleotide composition of aligned reads were analyzed. Nucleotide positions with an identity of ≥75% were considered. Samtools mpileup (Li et al., 2009) and in-house php scripts were used.

The authors apologize for this error and state that this does not change the scientific conclusions of the article in any way.

The original article was updated with the corrections.

## Conflict of interest statement

The authors declare that the research was conducted in the absence of any commercial or financial relationships that could be construed as a potential conflict of interest.
