# Supplementary material for: Identification of Rare PB2-D701N Mutation from a Patient with Severe Influenza: Contribution of the PB2-D701N Mutation to the Pathogenicity of Human Influenza
Source: Front Microbiol. 2017 Apr 3;8:575. doi: 10.3389/fmicb.2017.00575 (PMC5376584; doi:10.3389/fmicb.2017.00575)
Supplement: Supplementary file 1 [file Table_1.pdf]

Supplementary Table 1. NCBI database accession number of all viral isolates bearing PB2-701N

Human H1N1 Isolates from A(H1N1)pdm09-linage	Identification number
A/Aragon/270/2014	KY887997 (GISAID EPI_ISL_257783)
A/Uganda/MUWRP-111/2009	AHY84572
A/Wisconsin/51/2009	AGI53606
A/Singapore/GP3828/2010	AFP35784
**Human H1N1 isolates from swine origin**	**Identification number**
A/Jiangsu/ALS1/2011	ADW01404
A/Jiangsu/1/2011	AGN69333
A/Switzerland/9356/2009	ADU25464
A/Switzerland/5165/2010	ADU25485
